# Enriched Environment and Exercise Enhance Stem Cell Therapy for Stroke, Parkinson’s Disease, and Huntington’s Disease

**DOI:** 10.3389/fcell.2022.798826

**Published:** 2022-03-03

**Authors:** Reed Berlet, Dorothy Anne Galang Cabantan, Daniel Gonzales-Portillo, Cesar V. Borlongan

**Affiliations:** ^1^ Chicago Medical School at Rosalind Franklin University of Medicine and Science, North Chicago, IL, United States; ^2^ Michigan State University College of Osteopathic Medicine, East Lansing, MI, United States; ^3^ University of Florida, Gainesville, FL, United States; ^4^ Center of Excellence for Aging and Brain Repair, Morsani College of Medicine, University of South Florida, Tampa, FL, United States; ^5^ Department of Neurosurgery and Brain Repair, Morsani College of Medicine, University of South Florida, Tampa, FL, United States

**Keywords:** stem cell, rehabiliatation, enriched enviroment, exercise, combination therapy, Parkinson’s disease, huntingtons’s disease, stroke

## Abstract

Stem cells, specifically embryonic stem cells (ESCs), mesenchymal stem cells (MSCs), induced pluripotent stem cells (IPSCs), and neural progenitor stem cells (NSCs), are a possible treatment for stroke, Parkinson’s disease (PD), and Huntington’s disease (HD). Current preclinical data suggest stem cell transplantation is a potential treatment for these chronic conditions that lack effective long-term treatment options. Finding treatments with a wider therapeutic window and harnessing a disease-modifying approach will likely improve clinical outcomes. The overarching concept of stem cell therapy entails the use of immature cells, while key in recapitulating brain development and presents the challenge of young grafted cells forming neural circuitry with the mature host brain cells. To this end, exploring strategies designed to nurture graft-host integration will likely enhance the reconstruction of the elusive neural circuitry. Enriched environment (EE) and exercise facilitate stem cell graft-host reconstruction of neural circuitry. It may involve at least a two-pronged mechanism whereby EE and exercise create a conducive microenvironment in the host brain, allowing the newly transplanted cells to survive, proliferate, and differentiate into neural cells; vice versa, EE and exercise may also train the transplanted immature cells to learn the neurochemical, physiological, and anatomical signals in the brain towards better functional graft-host connectivity.

## 1 Introduction

Neurological diseases such as stroke, Parkinson’s disease (PD), and Huntington’s disease (HD) remain significant contributors to long-term disability and financial burden for patients worldwide. Despite decades of research into their underlying pathology and potential therapeutic targets, limited treatment options exist for these conditions. Regenerative medicine offers a promising intervention for these pathologies, but still faces significant hurdles to overcome. Of these issues, the ability to use stem cell-derived neural progenitors and integrate them fully into existing neural circuits to provide a more functional benefit while also enhancing the general outcome of transplantation has possible solutions; exercise and rehabilitation. This paper aims to discuss some of the current therapeutic uses of regenerative medicine and how rehabilitation strategies may increase the efficacy of stem cell grafts for long-term recovery. Stroke, PD, and HD were explicitly chosen due to the relative abundance of literature about exercise, EE, and stem cells (Table 1).

**TABLE 1:** The pathophysiology and common treatment options for ischemic stroke, Parkinson's disease, and Huntington's disease.

Condition	Pathophysiology	Treatment options
Ischemic Stroke	Reduction in cerebral blood flow from occlusion leads to	Acute Treatment options
	• Glucose and oxygen deprivation	• Alteplase (tPa)—Initiates local fibrinolysis by binding to fibrin in a blood clot and converts plasminogen to plasmin
	• ATP depletion	○ Must be initiated within 4.5 h of onset
	• Ionic concentration disequilibrium	• Mechanical thrombectomy - indicated after large artery occlusion in the anterior circulation and must be performed within 24 h of onset (Khaku and Tadi, 2021)
	○ Increased intracellular calcium and sodium	
	○ increased extracellular potassium	
	• Increased lactate	
	• Acidosis	
	• Accumulation of ROS	
	• Intracellular accumulation of water	
	• Activation of proteolytic processes	
	• Glutamate excitotoxicity	
	• Cell apoptosis and necrosis	
	• Disruption of the BBB	
	Focal or global deficits are based on the location and magnitude of the ischemic event	
	Caplan (2009); Markus. (2004); Doyle et al. (2008)	
Parkinson’s disease	Genetic disposition, idiopathic causes, and environmental factors lead to apoptosis of dopaminergic neurons by	Current treatment options for managing symptoms include
	• Excitotoxicity	• Monoamine oxidase type B (MAO B) inhibitors
	• Inflammation	• Amantadine
	• Mitochondrial dysfunction	• Levodopa
	• Neurotrophic failure	• Dopaminergic agonists
	• Oxidative stress	• Deep Brain Stimulation (DBS)
	• Proteasomal dysfunction	• Anticholinergics
	• Protein aggregation (A-synuclein/lewy body formation)	Connolly and Lang (2014), Ives et al. (2004), Schwab et al. (1972), Rizek et al. (2016)
	Depletion of dopamine due to neuronal loss in the basal ganglia disrupts connections with the motor cortex and thalamus. These deficits manifest as motor and nonmotor symptoms	
	Moore et al. (2005), Bergman and Deuschl (2002), Rizek et al. (2016)	
Huntington’s disease	A toxic gain-of-function trinucleotide repeat expansion of CAG within the coding region of the IT15 gene for the protein huntingtin on chromosome 4 leads to an elongated stretch of glutamine and eventual cell death of medium-sized spiny neurons (MSNs) in the striatum and cortex. The death of these MSNs that utilize GABA for neurotransmission leads to a lack of inhibition in basal ganglia circuitry. The disease manifests as chorea, cognitive disturbances, mood disorders, dystonia, rigidity, tics, myoclonus, and more	Current treatment options for managing symptoms include
	Some possible cellular mechanisms of dysfunction by mutant huntingtin include	• VMAT2 Inhibitors
	• Transcription disruption	• Muscarinic antagonists
	• Activation of proteases	• First-generation antipsychotics
	• Reduction in protein degradation	• Second-generation antipsychotics
	• Interference with axonal transport	• Benzodiazepines
	• disruption of synaptic transmission	• Anticonvulsants
	• Interference with wild type huntingtin	• Amantadine
	• Alteration of tau splicing and tau hyperphosphorylation	• Cannabinoids
	• Impaired nuclear-cytoplasmic transport	Nance et al. (2011), Armstrong et al. (2012)
	• Excitotoxicity	
	• Oxidative stress	
	• Apoptosis	
	• Metabolic dysfunction	
	• Impaired neuroblast migration	
	Raymond et al. (2011), Walker (2007), Gil & Rego, (2008), Dayalu and Albin (2015)	

Regenerative medicine utilizes a multitude of progenitor cell lines that vary based on biological origin, differentiation, advantages, and disadvantages (Table 2). Embryonic Stem Cells (ESCs) are undifferentiated pluripotent cells derived from mammalian blastocysts and can differentiate into any cell of all three germ layers, but pose major ethical concerns and have potential for immune rejection (Niwa et al., 2000; Chew et al., 2005). Induced pluripotent stem cells (IPSCs) are generated from adult somatic cells and bypass the ethical and immunogenicity concerns seen in ESCs, but may be tumorigenic, and it can be challenging to produce specific neurons with high purity (Okita et al., 2007; Qin et al., 2013; Doi et al., 2014; Yu et al., 2014). Nonetheless, IPSCs are a popular cell line for studying neurodegenerative diseases such as PD and HD. IPSCs can be differentiated into disease-specific neurons (ex. dopaminergic neurons in PD) that reflect the donor’s genetic markers and provide insight into changes in neurite morphology during disease progression (Park et al., 2008; de Rus Jacquet, 2019; Simmnacher et al., 2020). Mesenchymal stem cells (MSCs) are multipotent cells that secrete therapeutic substances and may migrate to the site of injury and putatively differentiate into the neural lineage (Pittenger et al., 1999; Ries et al., 2007; Waterman et al., 2010). MSCs’ anti-inflammatory effects make this an effective cell type for transplantation in ischemic stroke; indeed, MSC transplantation improves neurologic function in stroke models (Lee et al., 2016; Stonesifer et al., 2017). However, human MSCs exhibit distinct stemness properties from murine MSCs; thereby, it is important to consider the species source of the cells (Bonab et al., 2006; Miura et al., 2006). MSCs are routinely harvested from the bone marrow, and referred to as bone-marrow-derived derived MSCs (BM-MSCs). Controversy exists on the migration of BM-MSCs across the BBB and thereafter differentiating into neural cells. While a few peripherally transplanted BM-MSCs may reach the brain, most of the grafted cells lodged into inflammation-riches peripheral organs, such as the spleen, thymus, and cervical lymph nodes. Accordingly, whereas a few BM-MSC may differentiate into neural lineage and may accompany functional recovery of transplant recipients, the most likely regenerative mechanism entails the bystander effects *via* secretion of growth factors and other therapeutic substances. Nonetheless, these multi-pronged regenerative processes, including cell replacement and bystander effects, may aid in the cerebrovascular restoration post-ischemia (Eckert et al., 2013; Chen et al., 2015; Shichinohe et al., 2015; Stonesifer et al., 2017; Li et al., 2021). Finally, neural stem cells (NSCs) are primarily located in the subventricular zone (SVZ) and subgranular zone (SGZ) zones of the brain and can be derived from adults (Eriksson et al., 1998; Doetsch et al., 1999). NSCs are utilized for studying stroke, neurodegenerative disease, and trauma and can be produced *in vivo* and *in vitro*. They bypass ethical and immunogenic concerns and secrete growth factors promoting neuronal survival, but may promote tumor growth and can be difficult to isolate (Laywell et al., 2007; Amariglio et al., 2009; Bacigaluppi et al., 2009). Recent advances in regenerative medicine also include organoids and directly induced neurons. Organoids are *in vitro* 3D models grown from either pluripotent embryonic stem cells or adult stem cells (Grassi et al., 2019). Such a 3D model reflects the complex tissue organization of the host tissue that a single cell layer culture cannot and can generate from all three germ layers if derived from ESCs or IPSCs. More specifically, brain tissue organoids are made from neural progenitor cells (NPCs), which can differentiate into neurons and astrocytes (Rossi et al., 2018; Grassi et al., 2019; Corrò et al., 2020). Directly induced neurons may be used for studying age-related neurodegenerative diseases, where transcription factors and chemical signals are used to convert terminally differentiated cells across stages of aging (Mertens et al., 2018).

**TABLE 2:** Common stem cells and neural-progenitor cell types used for the experimental treatment of ischemic stroke, Parkinson's disease, and Huntington's disease.

Neural progenitor cell types			
Type	Description	Advantages	Disadvantages
Embryonic Stem Cells (ESCs)	Undifferentiated pluripotent cells derived from mammalian blastocysts; Chew et al. (2005)	• Can differentiate into any cell of all three germ layers; Niwa et al. (2000)	• Major ethical concerns due to these cells deriving from human blastocysts
			• Potential immune system rejection
Induced Pluripotent Stem Cells (IPSCs)	Pluripotent cells that are generated from adult somatic cells that can differentiate into any other cell; Qin et al. (2013)	• Very easy to produce ethically. Since they can be derived from adult cells, it also can bypass issues of immunogenicity; Yu et al. (2014)	• Tumorigenic risk; Okita et al. (2007)
		• Can differentiate into any kind of cell	• Difficulties with producing specific neurons with high purity; Doi et al. (2014)
Mesenchymal Stem Cells (MSCs)	Multipotent cells with the ability to differentiate into mesodermally derived cells (Pittenger et al., 1999)	• Can be proinflammatory or anti-inflammatory (Waterman et al., 2010)	• Human MSCs have critical differences to murine MSCs, making animal models less useful; Miura et al. (2006)
		• Responsive to the microenvironment can give it a “homing” ability to the site of injury (Ries et al., 2007)	• Difficult to produce *in vitro* because they can age and lose differentiation abilities (Bonab et al., 2006)
Neural Stem Cells (NSCs)	Located primarily in the SVZ and SGZ, responsible for development and upkeep of the brain; Doetsch et al. (1999); Can be derived from the adult themself; Eriksson et al. (1998)	• Bypass ethical issues and immunogenicity considerations	• Tumorigenic risk; Amariglio et al. (2009)
		• Can be produced *in vivo* and *in vitro*; Laywell et al. (2007)	• Difficult to isolate
		• Secrete growth factors which promote survival of surrounding neurons; Bacigaluppi et al. (2009)	—

Environmental enrichment (EE) is the addition of physical, sensory, or social stimulation into an animal’s environment. In experimental models, EE exerts a positive role in promoting regeneration, neurogenesis, and CNS remodeling. Exposure prevents relapse, enhances attention performance, reduces anxiety levels during development, prevents DNA methylation changes brought by aging, and enhances neurogenesis by increasing NPCs (Korkhin et al., 2020; Zocher et al., 2021; Zorzin et al., 2021; Pintori et al., 2022). Similarly, exercise promotes positive effects on neurogenesis, trophic factor secretion, motor recovery following ischemia, cognitive and motor function, and neuroprotection (Pang et al., 2006; Mang et al., 2013; Tsai et al., 2019). Harnessing these beneficial effects of EE and exercise in combination with stem cell therapy holds significant promise for advancing treatment in stroke, PD, and HD, where therapeutic options remain limited.

### 1.1 Stroke

Stroke is the second leading cause of death globally, costing patients in the United States nearly $103.5 billion in 2016 (Girotra et al., 2020). Approximately 87% of strokes are ischemic, where thrombotic or embolic events disrupt blood supply and deprive neurons of oxygen, leading to acute neuronal injury and death (Kuriakose and Xiao, 2020). The affected brain tissue with <20% of cerebral blood flow is defined as the ischemic core, where hypoxia results in irreversible damage and apoptosis within minutes to hours following the insult. Surrounding this core is the ischemic penumbra, where the neuronal function may still be recovered by restoring blood flow to the region (Anrather and Iadecola, 2016). Ischemic injury induces neuroinflammation, oxidative stress, and excitotoxicity, which contribute to underlying stroke pathology and impede healing (Kuriakose and Xiao, 2020; Anrather and Iadecola, 2016). Current reperfusion strategies utilize thrombolytic agents such as tissue plasminogen activator (tPA) or surgical intervention via mechanical thrombectomy (Table 1). The recent DAWN trial showed that the therapeutic window for mechanical thrombectomy could be prolonged to 24 h in select ischemic cases (Khaku and Tadi, 2021). Interestingly, after the critical period, reperfusion by mechanical thrombectomy or tPa amplifies existing neuronal injury due to reactive oxygen species (ROS) and subsequent neuroinflammation (Mandalaneni et al., 2021). While these techniques confer therapeutic advantages, they may also lead to hemorrhage following ischemic reperfusion (Nogueira et al., 2018). Additionally, tPA’s narrow therapeutic window, short half-life, and poor penetration of large clots warrant further investigation into effective therapies for stroke (Zamanlu et al., 2018).

### 1.2 Parkinson’s Disease

**FIGURE 1:**
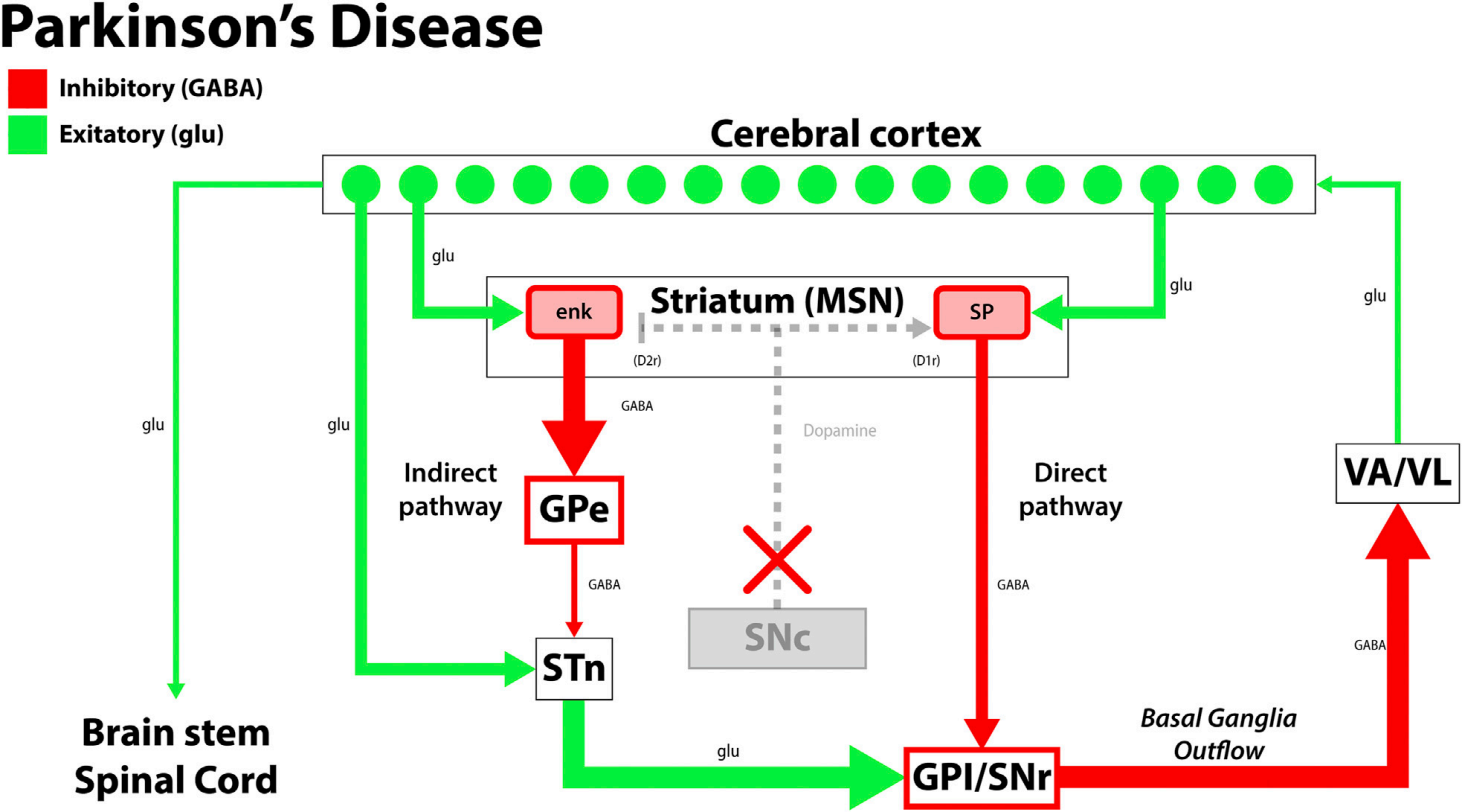
The disruption in the functional circuit in Parkinson’s disease. In PD, loss of the dopaminergic neurons of the substantia nigra pars compacta (SNc) leads to increased output by the indirect pathway and less motor output. The loss of dopaminergic neurons also impacts the direct pathway, increasing the circuit’s inhibition on the thalamus (VA/VL). Green arrows signify glutamatergic excitatory neurons and red arrows signify GABA expressing inhibitory neurons. enk, enkephalin; SP, Substance P; GPe, globus pallidus, external segment; GPi, globus pallidus, internal segment; SNc, substantia nigra pars compacta; SNr, substantia nigra pars reticulata; STN, subthalamic nucleus; VA/VL, ventral anterior/ventral lateral nucleus of the thalamus; D1r, D1 dopaminergic Gs coupled receptor; D2r dopaminergic Gi coupled receptor. Adapted from (McGregor and Nelson, 2019).

PD is the second most common age-related neurodegenerative disease globally, characterized by the death of dopaminergic neurons in the substantia nigra. This complex disease process leads to motor and non-motor symptoms, with neurodegeneration implicating the central, autonomic, and enteric nervous systems (Table 1) (Reeve et al., 2014; Simon et al., 2020; Mak et al., 2017; Stoker and Barker, 2020). Classic motor manifestations include cogwheel rigidity, an asymmetric resting tremor, bradykinesia, and non-motor features such as depression, dementia, and rapid-eye-movement (REM) sleep disorders that precede motor symptoms and contribute to cognitive decline (Pantcheva et al., 2015; Hayes et al., 2019; Simon et al., 2020; Balestrino and Schapira, 2020). Current therapeutic approaches for PD focus on replenishing dopamine stores, as in the case of levodopa, which is considered the gold standard treatment for PD. Levodopa is the biological precursor to dopamine that improves motor function early in the disease process, but may cause dyskinesias and fluctuating periods of enhanced and impaired motor function due to alterations in extra-striatal dopamine (Stoker and Barker, 2020; Hayes et al., 2019). In patients with mid-stage and advanced-stage PD, deep brain stimulation (DBS) confers therapeutic benefit by alleviating dyskinesias and medication-refractory tremors. Surgically implanted electrodes stimulate the subthalamic nucleus and globus pallidus internus, and stimulation parameters are tailored to the patient’s clinical state (Figure 1). While DBS is a well-established treatment option for PD, adverse effects may include dyskinesias, imbalance, and dysarthria, which should be weighed against its therapeutic benefits (Habets et al., 2018; Hayes et al., 2019; Stoker & Barker 2020).

### 1.3 Huntington’s Disease

**FIGURE 2:**
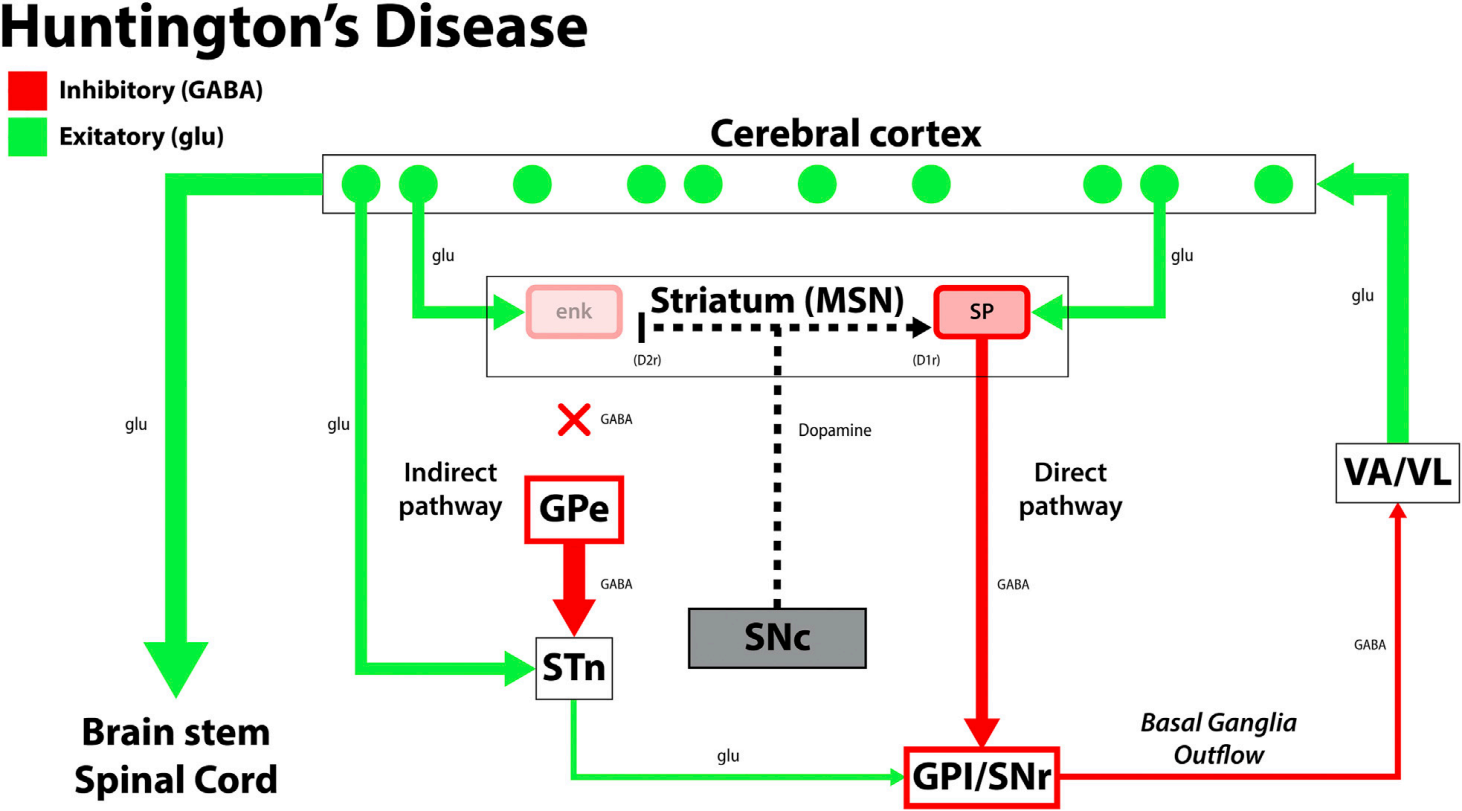
The disruption in the functional circuit in Huntington’s disease. Cerebral cortex atrophy and loss of the indirect pathway leads to more motor output and results in the choreiform movements seen in HD. Green arrows signify glutamatergic excitatory neurons and red arrows signify GABA expressing inhibitory neurons. enk, enkephalin; SP, Substance P; GPe, globus pallidus, external segment; GPi, globus pallidus, internal segment; SNc, substantia nigra pars compacta; SNr, substantia nigra pars reticulata; STN, subthalamic nucleus; VA/VL, ventral anterior/ventral lateral nucleus of the thalamus; D1r, D1 dopaminergic Gs coupled receptor; D2r dopaminergic Gi coupled receptor. Adapted from (Essa et al., 2019).

HD is a neurodegenerative disorder characterized by trinucleotide (CAG) repeat expansions in the huntingtin gene (HTT) on chromosome 4. It is inherited in an autosomal dominant manner, with genetic anticipation creating longer CAG expansions via paternal transmission. This mutation leads to a degeneration of GABAergic medium spiny neurons (MSNs) in the basal ganglia, disrupting neural circuitry and associated symptomology (Figure 2). Individuals with more repeats experience the disease earlier and progress more rapidly than those with fewer repeats (Wyant et al., 2017; Kim et al., 2021). HD’s molecular pathology is complex, involving aggregate formation, transcriptional dysregulation, altered synaptic plasticity, and glial dysfunction (Jimenez-Sanchez et al., 2017). Clinically, patients present with choreiform movements, which may progress to dystonia, rigidity, and ataxia as the disease progresses. Psychiatric conditions such as depression, anxiety, and suicidal ideations are also associated with HD. Currently, treatments for HD are primarily palliative (Table 1). Tetrabenazine (TBZ) is a monoamine transporter inhibitor that effectively reduces dopamine levels but has a black-box warning for increased risk of depression and suicidal ideation from concurrent depletion of serotonin and norepinephrine (Wyant et al., 2017; Kumar et al., 2020).

The pathophysiology and genetic mechanisms of stroke, PD, and HD have been well-studied, yet current therapeutic options leave much to be desired. tPA, the FDA-approved fibrinolytic agent, is commonly used to treat acute ischemic stroke, has a narrow therapeutic window and short half-life. A mechanical thrombectomy may be performed when patients do not meet tPA criteria. However, procedural and post-operative complications such as access-site vessel and nerve damage, intracerebral hemorrhage, and pseudoaneurysm may occur (Balami et al., 2018). Current strategies for PD and HD remain palliative and primarily focus on dopamine replacement and monoamine depletion. Finding targeted therapies that harness a disease-modifying approach for these conditions and a wider therapeutic window in the case of stroke will likely improve clinical outcomes for patients.

## 2 Regenerative Medicine for Treating Central Nervous System Disorders

Regenerative medicine, specifically stem cell-derived neural progenitor therapy, offers promising therapeutic potential for treating these CNS disorders. These three pathologies exhibit cell death and prove to be good candidates for stem cell-derived neural progenitor therapy. This option provides cell regeneration, endogenous neural progenitor recruitment, anti-inflammatory properties, and circuitry reconstruction. While the exact mechanisms of stem cell-derived neural progenitors remain elusive, the therapeutic void for stroke persists and begs for new strategies for recovery.

### 2.1 Regenerative Medicine for Stroke

In stroke, mass cell death presents unique complications as it is the death of neurons and the neurovascular unit as a whole within the ischemic core. The neurovascular unit comprises neurons, astrocytes, endothelial cells of the blood-brain barrier (BBB), myocytes, pericytes, and extracellular matrix components (Yasuhara et al., 2006; Muoio et al., 2014). The rescue of neurons and the neurovascular unit in the ischemic penumbra is feasible if acted upon promptly and thoughtfully using the suitable stem cells, dosage, and delivery route (Borlongan, 2019). In models of ischemic stroke, transplanted stem cell-derived neural progenitors not only replace infarcted tissue, but are neuroprotective, combat neuroinflammation, promote angiogenesis, reduce glial scar formation, and recruit endogenous neural progenitors to the area of injury (Doeppner and Hermann, 2010; Stonesifer et al., 2017; Borlongan, 2019; Saft et al., 2020). The discovery of endogenous neural stem cells (NSCs) in the human brain led to a new avenue of experiments with stem cell-derived neural progenitors and the eventual discovery of the biobridge (Renolds and Weiss, 1992; Ming and Song, 2011). The biobridge is the concept that transplanted neural progenitors not only replace dying cells and mitigate aberrant inflammation via the bystander effect but guide native neural progenitor cells from the host’s neurogenic niches to the area of infarction as well (Tajiri et al., 2013). The bystander effect is the process where transplanted stem cells secrete therapeutic substances rather than differentiating into neural cells, and may involve rapid attenuation of inflammation by reducing expression of proinflammatory factors, decreasing microglial activation, and promoting BBB repair. This anti-inflammatory effect was displayed when human-induced pluripotent stem cell-derived neural stem cells (hiPSC-NSCs) were transplanted into rodents 24 h after the onset of stroke. By the 48-h period, these transplanted cells migrated to the area of infarction and downregulated aggravating molecules such as tumor necrosis factor-α (TNF- α), IL-6, IL-1β, monocyte chemotactic protein 1 (MCP-1), macrophage inflammatory protein 1a (MIP-1a), intracellular adhesion molecule 1 (ICAM-1), and vascular cell adhesion molecule 1 (VCAM-1) (Eckert et al., 2015). In addition to the bystander effect, the endogenous NSCs from the neurogenic niche essentially replace transplanted stem cells and recapitulate the secretion of anti-inflammatory cytokines, proteomes, and neurotrophic factors to mitigate the harsh environment and facilitate functional recovery (Crowley and Tajiri, 2017; Liska et al., 2017). Additionally, these progenitor cells can differentiate into tissue-specific neurons, astrocytes, and oligodendrocytes to result in more favorable outcomes (Corey et al., 2019; Zhang et al., 2020). However, successful integration into fully functional neurons persists as a problem for both transplanted and endogenous neural progenitors.

Entry of peripherally administered cells across the BBB remains a controversial topic. Grafted BM-MSCs may use upregulated P- and E-selectins after stroke to adhere to endothelial cells in the cerebral vasculature (Huang et al., 2000; Yilmaz et al., 2011). Additionally, the upregulation of VCAM-1 after stroke aided NSC adhesion in another study, further highlighting the ability of these cells to use selectin mediated rolling and integrin associated adhesion to penetrate the BBB (Guzman et al., 2008). MSC adhesion to the cerebral endothelial cells led to secretion of CXCl-11 which binds CXCR-3 and increases BBB permeability through the ERK1/2 signaling pathway (Feng et al., 2014). Interestingly, MSCs have also shown to reduce BBB disruption by inhibiting matrix metallopeptidase 9 (MMP-9) and decreasing ICAM-1 (Cheng et al., 2018). This discrepancy may be due to the different stem cells used and the different stages of these stem cells, which needs to be further studied (Zhou et al., 2021). There seems to be a two-pronged mechanism where stem cells will use BBB permeability to extravasate and migrate to the injured area and then begin to secrete growth factors and therapeutic substances through the bystander effect (Ao et al., 2018).

### 2.2 Regenerative Medicine for Parkinson’s Disease

Preclinical studies prove the viability of stem cell-derived neural progenitors in PD treatment. However, clinical trials display findings that show incongruities between preclinical and clinical results (Yasuhara et al., 2006; Kirkeby et al., 2017). Like in stroke, translating results from the preclinical to clinical settings poses barriers, including which type of stem cells to use, the route of administration, the timing, the induction of cell differentiation, and facilitating transplanted cell survival. Regardless, stem cells have proven to be an effective treatment in rodent models due to their ability to differentiate into tissue-specific cells, secrete neuroprotective factors, induce endogenous repair mechanisms, recruit endogenous neural progenitors, modulate immune processes, increase survival of existing cell populations, and lead to a more functional recovery (Yasuhara et al., 2017; Takahashi, 2018; Yasuhara et al., 2006; Tajiri, Yasuhara, Shingo et al., 2010). Current stem cell therapies utilize MSCs, NSCs, ESCs, and IPSCs (Table 2). ESCs are pluripotent, but create ethical concerns, may trigger tumorigenesis, and stand frequent immunological rejection (Bradley et al., 2002; Bieberich et al., 2004). Compared with other artificially-induced neural progenitors, IPSC cell lines can be differentiated into dopaminergic neurons *in vitro* before transplantation, genetically tailored to match the dysfunctional transcription factor responsible for the patient’s phenotype, and permit patient-specific human leukocyte antigen (HLA) matches to reduce immune incompatibility. The use of IPSC-derived neural progenitor cells additionally circumvents a crucial ethical dilemma in stem cell research, as embryos are not utilized in this technology. (Takahashi and Yamanaka, 2006; Morizane et al., 2017; Playne and Connor, 2017; Stoddard-Bennett and Pera, 2019).

After successful stem cell therapy in PD, an overarching issue still exists, specifically the full integration of the immature cells into the host’s neural circuitry (Weiss et al., 2006). In one preclinical study, human NSCs cloned by v-myc gene transfer (HB1.F3 cells), were transplanted into the 6-hydroxydopamine-lesioned striatum of rats. The treated rats showed significant recovery of parkinsonian symptoms in comparison to controls. The lesioned rats exhibited a nearly complete restoration of spontaneous motor activity after intracranial transplantation and a small fraction of the neurons were positive for tyrosine hydroxylase (TH) along the nigrostriatal pathway, indicating a successful integration into tissue-specific neurons. However, many of the neurons remained nestin-positive or lacked TH in the presence of MAP2, suggesting many of the grafted cells remained immature or mature with a lack of complete differentiation into dopaminergic neurons, respectively (Yasuhara et al., 2006). Despite lack of dopaminergic differentiation, the transplanted NSCs exhibited neuroprotective effects. Studies have also shown improved Parkinson’s symptomatology even with minimal dopaminergic differentiation, suggesting that the bystander effects may play a more substantial role as grafts improve functional recovery after the loss of dopaminergic neurons in the substantia nigra (Jung et al., 2004; Goldman, 2005; Rafuse et al., 2005; Yasuhara et al., 2006). In addition to increased secretion of neurotrophic factors such as human recombinant stem cell factor (SCF) in both the neural progenitor cells of the SVZ and the grafted HB1.F3 cells, the study also observed increases in endogenous neural stem cell production and preservation of existing neuronal circuitry and dopaminergic neurons (Yasuhara et al., 2006). This study suggest the efficacy of the stem cell treatment in early PD can ameliorate the functional loss associated with the disease pathology with minimal or even lack of neural differentiation and maturation. In another study by Kirkeby et al., human ESC derived neural progenitor cells were transplanted into the striatum of 6-hydroxydopamine (6-OHDA) lesioned rats to assess their ability to obtain regional specification and survive. Tumor-free grafts were able to proliferate and reach full maturity, displaying an affinity for axonal outgrowth (Kirkeby et al., 2012). Another issue that regenerative medicine faces is the threat of inflammation after injection of stem cells in PD models. One investigation administered ESC derived neuronal cells into the brains of rats that received surgery to cause brain injury. At the 7-week mark after implantation, histological analysis discovered extensive loss of the grafts at the ipsilateral site of only one animal. Further analysis revealed that macrophages infiltrated the graft site at a high concentration and activated astrogliosis despite the administration of immunosuppressants (Molcanyi et al., 2007).

With a compelling body of evidence, strategies adjunct to stem cell therapy have been designed to enhance neuronal survival, development, and differentiation into fully functional dopaminergic neurons capable of mitigating PD deficits. Two potential strategies to achieve this improved graft function involve exercise and EE, as discussed in section 3.2 below.

#### 2.2.1 Regenerative Medicine for Parkinson’s Disease in Clinical Trials

In a 2001 double-blind open clinical trial, embryonic dopamine neurons were surgically transplanted in 20 out of 39 patients with PD. Imaging and postmortem studies showed a significant increase in dopaminergic neurons and neuronal differentiation in 17 out of 20 patients. Only 15% of the patients reported a recurrence of dystonia and dyskinesias after a one-year follow-up. The results proved human embryonic dopamine neuron transplants survive and lead to functional recovery in younger patients compared to older (over 60). This difference between younger and older patient recovery is likely due to a lack of robust neural plasticity in the older patients (Freed et al., 2001). While the use of embryonic grafts remains controversial, these findings indicate a viable option in the treatment of early PD. However, the lack of neuroplasticity in the aged brain may be overcome by specific interventions such as exercise and enriched environments (EE) to reinforce the circuitry provided by cell transplantation. The TRANSEURO clinical trials are ongoing but have problems collecting the human fetal ventral mesencephalic tissue needed for transplantation and have subsequently stopped. This barrier, alongside ethical dilemmas, has led to researchers searching for an alternative tissue supply (Barker et al., 2017). In response to this need, momentum has continued after the trials by Freed et al. and TRANSEURO despite the ethical concerns concerning embryonic stem cell use with the modern use of pluripotent stem cell (PSC)-derived dopamine neurons in humans (Barker et al., 2017).

### 2.3 Regenerative Medicine for Huntington’s Disease

HD continues to be an incurable disease, and current therapeutic options primarily focus on symptomatic treatment. Significant barriers to HD therapy include limited knowledge on molecular mechanisms implicated by the Huntington protein (HTT), extra-striatal atrophy throughout disease progression, and its broad impact on multiple systems, thus necessitating a systemic approach to treatment. Fortunately, advances in cell reprogramming and gene therapy offer promising potential to regenerate the damaged circuitry in the striatum and improve motor outcomes (Zheng and Kozloski, 2017; Cho et al., 2019; Monk and Connor, 2020). Cho and colleagues utilized gene therapy and neural progenitor cell transplantation to improve the motor deficits seen in the HD phenotype. The authors genetically corrected a mutant HTT NPC line and produced a viable cell therapy product that, like the WT NPC line, improved motor performance and lifespan when grafted into the striatum of HD mice (Cho et al., 2019). However, it is worth noting that inducing genetic changes before transplantation may introduce genetic instability (i.e., malfunctions in DNA repair, spindle formation, and telomere abnormalities) that can collectively contribute to tumor formation (Ross et al., 2011). Nonetheless, stem cell-derived neural progenitor cell transplantation stands as a promising therapeutic tool for HD.

### 2.4 The Need for Enhanced Graft-Host Integration and Function

Altogether, the developments in stem cell research for these three pathologies offer an exciting new avenue of possible subacute and chronic treatments. However, one problem remains, specifically, the use of immature cells in a previously integrated neural network. The use of immature cells, while key in recapitulating brain development, presents the challenge of young grafted cells forming neural circuitry with the mature host brain cells. To this end, exploring strategies designed to nurture graft-host integration will likely enhance the reconstruction of the elusive neural circuitry. The subsequent section discusses the use of EE/exercise in stem cell therapy-induced repair of the neural circuitry after stroke, PD, and HD.

## 3 Enriched Environment/Exercise and Pathology

Environmental enrichment and exercise are validated animal models for rehabilitation that can be measured through behavioral assessments (Huo et al., 2021). Clinical rehabilitation strategies vary based on subjective criteria such as patient characteristics, clinician preferences, and preferred outcomes. This makes it hard to standardize and attribute preferable outcomes to specific strategies (Huo et al., 2021). However, it is well known that rehabilitation and exercise lead to better functional outcomes, but the mechanisms by which they accomplish this and the effective period for intervention can be elusive. Recent findings in understanding motor learning, neuroplasticity, and functional recovery have led researchers to dive deeper into possible mechanisms by which EE and exercise lead to better outcomes and how they may be used in conjunction with other treatments and interventions. Here we will evaluate the preclinical data for stroke, PD, and HD regarding EE and exercise and show possible mechanisms of recovery that are used in combination therapies and can be further used in translational studies.

### 3.1 Enriched Environment/Exercise and Stroke

EE and exercise have been well studied in the context of stroke. EE strategies lead to increased gross neuroglia, sensorimotor function, spatial learning, and memory. However, outcomes are primarily based on the animal’s activity (Zhan et al., 2020). A three-phase paradigm has been proposed for the evolution of the EE throughout the healing process in an ischemic stroke rat model. Rats were initially exposed to social interaction, voluntary exercise, and small cabins for the first 2–7 days. They were then moved to a two-layer cage with more environmental interaction such as tunnels, swing boards, ladders, and balance beams for days 8–14. The third and final phase consisted of a three-layered cage with increasing slopes of the ladders and balance beams as well as floating cabins for days 15–30. The study showed significant restorative benefits with increased survival of neurons in the striatum and cortex, improved cerebral blood flow, increased angiogenesis, increased endogenous progenitor cell proliferation, increased endogenous neuronal differentiation in the ischemic regions, increased axonal guidance proteins, and reduced ischemic BBB capillary damage compared to standard housing rats (Zhan et al., 2020). Many molecular mechanisms induced by EE supported these incredible findings. Angiogenesis and the rescue of the neurovascular unit were portrayed through signaling pathways such as phosphorylated PI3K, AKT, and GSK-3, but reduced phosphorylated β-catenin. In tandem with modified expressions of vascular endothelial growth factor (VEGF), Angiopoietin-1 **(**Ang-1), and Angiopoietin-2 **(**Ang-2), the benefit from a modified EE program highlights the importance of rehabilitation in the recovery from stroke (Zhan et al., 2020). It has been shown that neuronal and endothelial cell proliferation happen together in the context of the neurovascular unit. The effects of EE on adult neurogenesis are replicable and have been displayed in many studies. Spatial learning significantly increased when rats were subjected to EE, and the positive results diminished when adding temozolomide, a drug that decreases neurogenesis (Garthe et al., 2016).

In addition to the combined benefits on neuronal proliferation, endogenous repair mechanisms, and neurogenesis, EE has proven to effectively induce neuroplasticity, cell maturation, genetic changes, and neuronal integration in host tissue (Kempermann, 2020). The theory posits that the actual benefits of EE come from the physical activity, social interaction, and cognitive stimulation elicited by the environment and must be assessed together. When studying hippocampal-dependent memory in rats, spatial enrichment modified the PKA dependence of long-term potentiation (Duffy et al., 2001). The study of genetic modifications in response to EE began in 2000 and has progressed (Rampon, 2000; Zhang et al., 2018). Enrichment modifies DNA methylation, including a regulator of adult neurogenesis, NeuroD1 (Zhang et al., 2018). Along with the genetic effects, neuronal maturation and integration are essential for portraying the benefits of EE. The experience of the animal leads to differing synaptogenesis and dendritic spine formation in the dentate gyrus (DG) during neurogenesis (Zhao et al., 2015). New neurons need stimulation to mature into tissue-specific neurons that are capable of remaining viable. Yu et al. demonstrated the neurovascular and behavioral outcomes after rehabilitation in an ischemic stroke model. EE in the form of climbing platforms, plastic tubes and tunnels, chains, and small boxes elicited neuroprotective effects and accelerated motor coordination recovery, progenitor cell integration, memory performance and more after partial middle cerebral artery occlusion. In addition, higher levels of CD31 were detected in the ischemic penumbra, revealing an increase in vascular differentiation (Yu et al., 2014).

Immunological studies also highlight the ability of EE in the form of nest boxes, fabric tubes, running wheels, and swing to change genetic factors and T Cell phenotype, leading to increases in IL-10 and IL-17 with subsequent decreases in IFN-γ (Rattazzi et al., 2016). This resolution of inflammation points to a broader immunological role of behavior and recovery. A review paper published in 2014 revealed the immunological changes were largely based on the EE in which the animals were placed. Physical exercise (PE), a form of EE, led to downregulation of toll-like receptors on macrophages and monocytes, reduced secretion of adipokines, modulation of hippocampal T cells, upregulation of mitogen-activated protein kinase phosphatase-1, and decreases in IL-1β and TNF-α. These immunomodulatory effects last after the stimulus and point to a long-term positive effect of EE and exercise (Singhal et al., 2014). A novel form of apoptosis, named pyroptosis, is a mechanism of cell death through membrane pore formation in response to infection and danger signals (Kuang et al., 2017). An experiment by Liu et al. displayed that an EE with ladders, platforms, swings, colorful balls, different-shaped wooden blocks, plastic tunnels, and a running wheel led to neuronal anti-pyroptosis through inhibition of Nuclear Factor kappa B (NF-kB) p-65 signaling pathway resulting in decreased levels of the inflammasome NLRP1 and NLRP3 after ischemia. Downstream, this pathway led to decreased levels of IL-1B, IL-18, and pyroptosis in neurons showing EE’s anti-inflammatory effects (Liu et al., 2021).

Exercise after an ischemic event has been combined with stem cells transplants (Table 3). However, it should be noted that timing for exercise, especially intense exercise, after an ischemic event is a determining factor for rehabilitative efficacy. Very early exercise interventions have a negative overall effect on the outcome of recovery after traumatic brain injury and may follow a similar pattern in stroke.

**TABLE 3:** Experimental studies for stroke with stem cell transplantation, an enriched environment, exercise, and a combination of enriched environment or exercise with stem cells.

Stroke			
Type of intervention (exercise/EE/stem cells/exercise + stem cells/EE + stem cells)	Title, Author, Year	Route of administration/stem cell type	Significant findings
Enriched Environment	The three-phase enriched environment paradigm promotes neurovascular restorative and prevents learning impairment after ischemic stroke in rats; Zhan et al. (2020)	Endogenous NPCs	MCAO rats were exposed to social interaction, voluntary PE, and small cabins in the first phase. In the second phase, rats were exposed ot a two-layered cage with more objects such as tunnels, swing boards, ladders and balance beams. The third phase consisted of a three-layer cage with increased complexity. The rats showed significant restorative benefits with increased survival of neurons in the striatum and cortex, improved cerebral blood flow, increased angiogenesis, increased endogenous progenitor cell proliferation, increased endogenous neuronal differentiation in the ischemic regions, increased axonal guidance proteins, and reduced ischemic BBB capillary damage compared to standard housing rats
Enriched Environment	Enriched Environment Attenuates Pyroptosis to Improve Functional Recovery After Cerebral Ischemia/Reperfusion Injury. (Liu J, Zheng J, Xu Y, et al., 2021)	N/A	MCAO rats subjected to cages containing ladders, platforms, swings, colorful balls, different-shaped wooden blocks, plastic tunnels, and a running wheel showed increased functional recovery, reduced infarct volume, and attenuated neuronal pyroptosis after reperfusion
Enriched Environment	Enriched Rehabilitative Training Promotes Improved Forelimb Motor Function and Enhanced Dendritic Growth after Focal Ischemic Injury; Biernaskie and Corbett, (2001)	N/A	Endothelin-1 induced ischemic stroke in rats with an EE consisting of shelves, plastic tubing, ladders, and rope showed increased dendritic complexity and length and improved the functional outcome
Enriched Environment	Enriched environment attenuates cell genesis in the subventricular zone after focal ischemia in mice and decreases migration of newborn cells to the striatum; Nygren et al. (2006)	Endogenous NSCs	MCAO rats subjected to an EE with a multilevel cage containing toys, ramps, and platforms for 3 h a day showed an increase in cell neurogenesis in the subventricular zone and dentate gyrus as well as improved functional outcome
Enriched Environment	Delayed exposure to environmental enrichment improves functional outcome after stroke; Tang et al. (2019)	N/A	MCAO rats subjected to EE with running wheels, climbing ladders, nest boxes, hammock, colored blocks and tunnels 5 days after infarct showed improved functional outcomes, increased survival and differentiation of hippocampal progenitor cells, increased synaptic density of mature neurons, and enhanced migration of NSCs from the SVZ.
Exercise	Treadmill exercise ameliorates focal cerebral ischemia/reperfusion-induced neurological deficit by promoting dendritic modification and synaptic plasticity via upregulating caveolin-1/VEGF signaling pathways; Xie et al. (2019)	N/A	MCAO rats subjected to treadmill exercise 2 days after the ischemic event had higher levels of dendritic and synaptic plasticity in the penumbra, improved neurological recovery, and reduced infarct volume
Exercise	Enforced PE promotes neurogenesis in the subgranular zone after focal cerebral ischemia Lee et al. (2008)	Endogenous NPCs	Enforced PE promotes endogenous neurogenesis in the subgranular zone (SGZ) after focal cerebral ischemia
Exercise	Early treadmill exercise increases macrophage migration inhibitory factor expression after cerebral ischemia/reperfusion; Chang et al. (2019)	N/A	MCAO rats subjected to forced exercise 2 days after ischemic event for 5 days showed improved motor and neuronal recovery and expressed higher levels of macrophage inhibiting factor and BDNF in the ischemic penumbra
Exercise	Exercise Intervention Promotes the Growth of Synapses and Regulates Neuroplasticity in Rats With Ischemic Stroke Through Exosomes	N/A	MCAO rats with exercise increased serum exosomes and improved synaptic growth, reduced infarct volume, and improved functional outcomes
Exercise	The Effects of Early Exercise on Motor, Sense, and Memory Recovery in Rats With Stroke; Yang et al. (2017)	N/A	MCAO rats subjected to moderate exercise within 48 h of stroke displayed significantly increased coordinated locomotor and spatial memory but not sensorimotor or vestibulomotor functions
Exercise	Physical exercise regulates neural stem cells proliferation and migration via SDF-1α/CXCR4 pathway in rats after ischemic stroke; Luo et al. (2014)	Endogenous NSCs	MCAO Rats showed increased functional recovery by increased endogenous NSC recruitment, improved migration from SVZ, and differentiation in the striatum
Exercise	Postischemic exercise attenuates whereas enriched environment has certain enhancing effects on lesion-induced subventricular zone activation in the adult rat; Komitova et al. (2005)	Endogenous NSCs	Exercise modulated the stroke-induced increase in NSC proliferation in the SVZ early after cortical infarction
Exercise	Different exercises can modulate the differentiation/maturation of neural stem/progenitor cells after photochemically induced focal cerebral infarction; Morishita et al. (2020)	Endogenous NSCs	Exercise improved neuronal maturation and increased generation of endogenous NSCs
Stem cell transplantation	Intravenous Grafts Of Amniotic Fluid-Derived Stem Cells Induce Endogenous Cell Proliferation and Attenuate Behavioral Deficits in Ischemic Stroke Rats; Tajiri et al. (2012)	Intracerebrally transplanted amniotic fluid-derived stem cells (AFS)	AFS cells aided endogenous NSCs to the infarction area via metalloproteinases (MMPs) and improved functional outcomes
Stem cell exosome transplantation	Enhancement of angiogenesis and neurogenesis by intracerebroventricular injection of secretome from human embryonic stem cell-derived mesenchymal stem cells in ischemic stroke model; Asgari Taei et al. (2021)	Intracerebrally/human ESC derived MSC exosomes	Human ESC derived MSC exosomes transplanted intracerebrally suppress inflammation, reduce cell death, promote angiogenesis, and stimulate neurogenesis
Stem cell transplantation	Activated Mesenchymal Stem Cells Induce Recovery Following Stroke Via Regulation of Inflammation and Oligodendrogenesis; Tobin et al. (2020)	IV/interferon-γ–activated MSCs and MSCs	Intravenous MSCs lowered inflammation molecules, ameliorated potentially toxic environments, and increased neurotrophic factor release, enabling both endogenous NSC survival and function
Enriched Environment and stem cell transplantation	Enriched environment enhances transplanted subventricular zone stem cell migration and functional recovery after stroke; Hicks et al. (2007)	Intracerebrally/NSCs	MCAO rats were given adult NSCs intracerebrally with an EE consisting of tubes, beams, shelves, rope, ladders, and a running wheel 7 days after stroke. Rats subjected to this environment had greater survival of SVZ stem cell transplants, greater migration to the infarction, and increased functional recovery
Exercise and stem cell transplantation	Treadmill exercise enhances therapeutic potency of transplanted bone mesenchymal stem cells in cerebral ischemic rats via anti-apoptotic effects; Zhang et al. (2015)	BM-MSCs	Treadmill exercise increases the therapeutic benefit of MSCs by improving neurological function and inhibiting the apoptosis of neurons and transplanted MSCs
Exercise and stem cell transplantation	Synergic Effects of Rehabilitation and Intravenous Infusion of Mesenchymal Stem Cells After Stroke in Rats (Sasaki et al., 2016)	IV/MSCs	Intravenous MSC with exercise decreased infarct volume, induced synaptogenesis, and increased functional outcomes compared to MSC transfusion alone
Exercise and stem cell transplantation	Effects of the combined treatment of bone marrow stromal cells with mild exercise and thyroid hormone on brain damage and apoptosis in a mouse focal cerebral ischemia model; Akhoundzadeh et al. (2017)	MSCs	Combined MSC and exercise led to a decrease in infarct volume and a decrease in apoptosis

### 3.2 Enriched Environment/Exercise and Parkinson’s Disease

EE and exercise may offer a solution to the maintenance and protection of dopaminergic neurons, evidenced through rat models. A study was conducted to investigate the neuroprotective functions of exercise in rats that were treated with 6-OHDA to emulate PD. A baseline was taken, with rats having access to an exercise wheel 2 weeks before the 6-OHDA injection. Rats would travel 3,361 ± 932 m/day before treatment, and after the brain lesions were induced they traveled 1,292 ± 770 m/day (Tsai et al., 2019). The study also assessed gait patterns and akinesia and took histological samples before and after the brain lesion. The immunohistochemistry analysis revealed that rats in the exercise condition displayed a significantly higher amount of dopaminergic neurons than the control group. Ultimately, the study’s results support the long-term neuroprotective effects of exercise through immunehistological results and behavioral assessments (Tsai et al., 2019). This research is further supported by Rezaee et al., who also investigated the effects of exercise in rats treated with 6-OHDA. This experiment assessed the expression of various genes that play critical roles during neurodegeneration and regeneration such as Ampk*,* Sirt1, Pgc1a. The study found that treadmill exercise for rats with the 6-OHDA injection significantly increased TH expression and brain derived neurotrophic factor (BDNF). This also contributed to the amelioration of the behavioral abnormalities of the rats in exercises like the apomorphine-induced rotations (Rezaee et al., 2020). The increased expression of neurotrophic factors and TH levels supports that exercise has neuroprotective properties that can play a critical role in the treatment of PD. EEs are another tool that shows promise in the treatment of PD, but these settings are not standardized, which emphasizes the importance of descriptive details for each study (Table 4). Jadavji et al. found that rats with PD that were placed in an EE with ladders, multileveled cages, and toys saw significant improvements in motor deficits such as skilled reaching, walking, and apomorphine-induced rotation compared to rats with PD in a standard housing environment. Furthermore, a histological assay that measured the amount of TH-positive cells found that rats in the EE group had a significantly higher amount than the standard environment group (Jadavji et al., 2006). However, an important consideration to these results is that the rats were placed in an EE before 6-OHDA treatment as well. This timeline for exposure to EEs, particularly before the onset of PD has been a point of interest because a relationship may exist. Jungling et al. conducted another study where they placed rat pups in an EE with larger cages and were exposed to intensive complex stimuli for 5 weeks after birth and then were placed in a regular environment afterward. They then received 6-OHDA injections at 3 months old. The study results demonstrated that the rats with early exposure to an EE performed significantly better on motor function tests and had less dopaminergic neuron loss after 6-OHDA treatment (Jungling et al., 2017). However, in this study, the maximal lesion of dopaminergic neurons was only 24%. Regardless, this investigation still supported the use of EEs as a preventative tool against PD (Jungling et al., 2017). Other studies have extended these findings to measure the effects of a combined intervention utilizing both EEs and exercise. An investigation by Pradhan incorporated both treatments through exercise video games with human subjects. This study recruited patients with mild PD, and selected games that specifically target PD-induced deficits such as balance, reflex responses, and cognitive engagement (Pradhan, 2019). The specific games were tailored to each subject based on a 1–10 rating of difficulty each participant provided after trialing each game. Participants performed physical assessments before and after the intervention that assessed motor deficits associated with PD such as a functional reach test, single limb stance, and gait speed. These results ultimately displayed behavioral improvements from baseline to post-intervention for the assessments that the games had targeted (Pradhan, 2019). These studies show firm support for practical applications of EEs and exercise as a treatment regimen for alleviating the debilitating symptoms of PD. Increasing expression of neurotrophic factors and protecting dopaminergic neurons from decay allow for improvements in motor skill deficits that originate from PD.

**TABLE 4:** Experimental studies for Parkinson's disease with stem cell transplantation, an enriched environment, exercise, and a combination of enriched environment or exercise with stem cells.

Parkinson’s disease				
Type of intervention	Title, Author, Year	Stem cell, method of administration	Significant findings	Other notes
Stem Cell Transplantation	Transplantation of embryonic dopamine neurons for severe Parkinson’s disease,; Freed et al. (2001)	ESCs, Cerebral Injection (Putamen)	Grafting embryonic stem cells into patients with PD demonstrated improved self-report scores on their symptoms for patients under 60 years old	The age of the patient played a significant role in the self-reporting, as patients over 60 did not have a significant amount of improvement in their self-reports
Stem Cell Transplantation	Generation of regionally specified neural progenitors and functional neurons from human embryonic stem cells under defined conditions,; Kirkeby et al. (2012)	ESCs, Cerebral Injection (Striatum)	Grafted neural progenitor cells that were derived from human ESCs into 6-OHDA lesion rats resulted in survival of all grafts to full maturation. Grafted cells were controlled by dose dependent activation of WNT signaling to arrange acquisition of regional phenotypes	Ventral midbrain specified cells formed the largest transplants. Experiment saw no tumor formation in any of the grafts, and saw significant proliferation in each of them
Stem Cell Transplantation	Human ESC-derived dopamine neurons show similar preclinical efficacy and potency to fetal neurons when grafted in a rat model of Parkinson’s disease, (Grealish et al., 2014	ESCs, Cerebral Injection (Striatum)	This study demonstrated that grafted ESCs induce the preservation and restoration of dopaminergic neurons and can lead to increased axonal outgrowth in 6-OHDA lesioned rats	The study also reported that the ESC-derived dopaminergic neurons were able to extensively reinnervate the striatum
Stem Cell Transplantation	Human Clinical-Grade Parthenogenetic ESC-Derived Dopaminergic Neurons Recover Locomotive Defects of Nonhuman Primate Models of Parkinson’s Disease, (Wang et al., 2018	ESCs, Cerebral Injection (Striatum)	This study demonstrated that ESCs can differentiate into dopaminergic neurons and ameliorate behavioral performance in monkeys	Study reported rapid recovery of monkeys after surgery, including the ones that did not receive the ESC injection
Stem Cell Transplantation	Prolonged maturation culture favors a reduction in the tumorigenicity and the dopaminergic function of human ESC-derived neural cells in a primate model of Parkinson’s disease,; Doi et al. (2012)	ESCs, Cerebral Injection (Striatum, Putament)	This study demonstrated that dopaminergic neurons could be generated from human ESCs in mice, but its growth is diminished by prolonged maturation in the culture. The study also used human ESCs in monkeys and found that undifferentiated human ESCs promote tumor formation, but prolonged maturation decreases that risk. The ESCs were also able to function as dopaminergic neurons in the MPTP-treated monkeys	Study reported that elimination of undifferentiated cells are not able to prevent the formation of neural masses
Stem Cell Transplantation	Functional engraftment of human ES cell-derived dopaminergic neurons enriched by coculture with telomerase-immortalized midbrain astrocytes,; Roy et al. (2006)	ESCs, Cerebral Injection	This experiment demonstrated that grafted ESCs in 6-OHDA lesioned rats benefitted from cocultures with mesencephalic astrocytes which were able to potentiate dopaminergic neurogenesis	The study reported that glia-mediated differentiation into dopaminergic neurons is region specific, only midbrain astrocytes were able to produce this type of neuron
Stem Cell Transplantation	Co-grafting astrocytes improves cell therapeutic outcomes in a Parkinson’s disease model; Song et al. (2018)	ESCs and IPSCs, Cerebral Injection (substantia nigra and median forebrain bundle)	This study demonstrated that NPC transplants in tandem with astrocytes can enhance the stem cells’ ability to differentiate into dopaminergic neurons. Co-grafting astrocytes also allowed for the grafted neurons to benefit from their paracrine effects of producing neurotrophic factors	Utilized hemiparkinsonian rat model. NPCs and astrocytes were derived from the ventral midbrain of rodent fetuses. Study also reported that in astrocytes that were modified to express Nurr1+Foxa2, and found that the NPCs near these astrocytes were more resistant to toxins like H2O2
Stem Cell Transplantation	Human iPS cell-derived dopaminergic neurons function in a primate Parkinson’s disease model; Kikuchi et al. (2017)	IPSCs, Cerebral Injection (Putamen)	The study demonstrated that IPSC derived dopaminergic neurons in MPTP-treated monkeys resulted in increased scores on the neurological rating scale	Animal model that utilized monkeys. The study screened for neural rosette-forming cells, which can contribute to tumors, but did not find these cells. The neurological rating scale consisted of a scoring system that assessed facial expression, head checking movement, spontaneous movement, movement in response to stimuli, tremor, postural instability, and gait. The study also reported no differences in recovery when the grafted cells came from healthy patients or ones with PD.
Stem Cell Transplantation	Personalized iPSC-Derived Dopamine Progenitor Cells for Parkinson’s Disease; Schweitzer et al. (2020)	IPSCs, Cerebral Injection (Putamen)	A patient that received injections in both hemispheres of the brain experienced a growing decline in parkinsonian symptoms on the UPDRS, part III. PET scans conducted during the study also revealed an improved 18F-DOPA PET signal near the graft site in the posterior putamen	The IPSCs were taken from the skin. The patient also had his levodopa prescription decline its daily dose by 6%
Stem Cell Transplantation	Human autologous iPSC-derived dopaminergic progenitors restore motor function in Parkinson’s disease models; Song et al. (2020)	IPSCs, Cerebral Injection (Striatum)	This study demonstrated how metabolism-regulating miRNAs can be used to efficiently program quality IPSCs. When these IPSCs were grafted into 6-OHDA lesioned rodents, the animals demonstrated ameliorated behavioral performance and preservation of dopaminergic neurons	The rotation behavior was fully rescued in all rodents16 weeks after the injection, whereas the control rodents did not experience any improvement
Stem Cell Transplantation	Successful function of autologous iPSC-derived dopamine neurons following transplantation in a non-human primate model of Parkinson’s disease; Hallett et al. (2015)	IPSCs, Cerebral Injection (Putamen)	The study demonstrated that a graft of dopaminergic neurons derived from IPSCs can integrate and survive for at least 2 years to improve motor function in a primate model	The study also reported extensive reinnervation of the denervated putamen
Stem Cell Transplantation	Intravenous administration of mesenchymal stem cells exerts therapeutic effects on parkinsonian model of rats: Focusing on neuroprotective effects of stromal cell-derived factor-1α; Wang et al. (2010)	MSCs, Intravenous Injection	Rats that received MSCs demonstrated behavioral amelioration and preserved neurons in the substantia nigra pars compacta	The *in vitro* portion of the study found that a secreted factor of the MSC suppressed cell death from 6-OHDA treatment
Stem Cell Transplantation	Multiple neurogenic and neuro-rescue effects of human mesenchymal stem cell after transplantation in an experimental model of Parkinson’s disease, Cova et al., 2010	MSCs, Cerebral Injection (Striatum)	The injection of human MSCs into rats resulted in enhanced neurogenesis and protection of dopaminergic neurons. The grafted cells also secreted multiple neurotrophic and angiogenic factors	Study reported that *in vivo*, none of the grafted cells were TH- or DAT-positive, indicating that the cells did not acquire the dopaminergic phenotype
Stem Cell Transplantation	Transplantation of human neural stem cells exerts neuroprotection in a rat model of Parkinson’s disease, Yasuhara et al., 2006	NSCs, Cerebral Injection (Intrastriatal)	Grafting fetal neural stem cells into rats treated with 6-OHDA resulted in behavioral amelioration, preservation of nigrostriatal dopaminergic neurons, and enhanced neurogenesis	The *in vitro* portion of this study demonstrated that NSCs have neuroprotective effects against 6-OHDA toxicity and secrete neurotrophic factors like SCF and BDNF.
Exercise	Long-term effects of exercise and physical therapy in people with Parkinson disease; Mak et al. (2017)	NSCs, Endogenous	Of all exercises, balance training’s beneficial effects endured the longest	A variety of physical activities were used that tested flexibility, strength, balance, coordination or aerobic training. The chosen exercises used at least 3 or more of these modalities
Exercise	Exercise exerts neuroprotective effects on Parkinson’s disease model of rats; Tajiri et al. (2010)	NSCs, endogenous	Exercise with 6-OHDA lesioned rats demonstrated behavioral amelioration, preservation of nigrostriatal neurons, enhanced migration of newborn neurons, and upregulation of neurotrophic factors	Utilized voluntary exercise in this study, however those with spinal cord injury received forced exercise, and both groups saw significant functional recovery
Exercise	Technology-Assisted Balance and Gait Training Reduces Falls in Patients With Parkinson’s Disease: A Randomized Controlled Trial With 12-Month Follow-up, (Shen and Mak, 2015)	N/A (Study investigated exercise and behavioral outcome)	This study demonstrated that balance and gait training that is enhanced by technological assistance via smart dancing mats, balance masters, and treadmills is effective in reducing falls for patients with PD.	The secondary outcome reported that the intervention group had a greater reduction in latency to postural response and greater increases in single-leg-stance times
Exercise	Tai Chi and Postural Stability in Patients with Parkinson’s Disease; Li et al. (2012)	N/A (Study investigated exercise and behavioral outcome)	This study demonstrated that Tai Chi is effective in reducing balance impairments, and as a result increased functional capacity and reduced falls	The study recruited participants with mild-to-moderate PD. The study also had other groups that consisted of resistance training, and another one that practiced stretching, however Tai Chi was the most effective of the three
Exercise	Effects of Tai Chi on balance and fall prevention in Parkinson’s disease: a randomized controlled trial; Gao et al. (2014)	N/A (Study investigated exercise and behavioral outcome)	The study demonstrated that Tai Chi could help prevent falls by improving balance	This study reported that Tai Chi did not see any difference compared to the non-intervention group in the Unified Parkinson’s Disease Rating Scale and Timed Up and Go
Exercise	Multi-dimensional balance training program improves balance and gait performance in people with Parkinson’s disease: A pragmatic randomized controlled trial with 12-month follow-up; Wong-Yu and Mak, (2015)	N/A (Study investigated exercise and behavioral outcome)	This study examined how balance training that blends indoor/outdoor settings can ameliorate weakened control systems from PD in order to prevent falls. The results demonstrated significant improvements in the Balance Evaluation Systems Test compared to baseline at the 12 month follow-up after this regimen	The control group’s regimen solely consisted of upper limb exercises
Exercise	Treadmill exercise elevates striatal dopamine D2 receptor binding potential in patients with early Parkinson’s disease, Fisher et al., 2013	NSCs, Endogenous	Exercise demonstrated behavioral amelioration and increased binding potential of the dopamine D2 receptor	Study utilized humans in a clinical trial. Study also reported that the participants demonstrated improved turning performance, but still had no significant change in their UPDRS scores
Exercise	Long-term voluntary physical exercise exerts neuroprotective effects and motor disturbance alleviation in a rat model of Parkinson’s disease, (Tsai et al., 2019)	NSCs, Endogenous	Exercise over an extended period of time attenuated motor decline and preserved dopaminergic neurons	Exercise for the rats occurred 2 weeks before 6-OHDA lesion and continued 8 weeks after the operation
Exercise	Exercise-Induced Neuroprotection in the 6-Hydroxydopamine Parkinson’s Disease Model,; Rezaee et al. (2020)	NSCs, Endogenous	The experiment demonstrated behavioral amelioration, increased expression of neurotrophic factors, and overall neuroprotective effects by exercise on a treadmill	Study utilized young rats. treatment. Rats that did not receive 6-OHDA treatment but also exercised saw increased mRNAs and proteins in the striatum
Enriched Environment	Effects of Postnatal Enriched Environment in a Model of Parkinson’s Disease in Adult Rats; Jungling et al. (2017)	NSCs, Endogenous	An enriched environment in newborn rat pups can protect dopaminergic neurons after 6-OHDA treatment	The enriched environment consisted of a larger cage with toys, tunnels, various rotating rods, with half of the toys being changed daily. After 5 weeks of this environment the rats were kept in a normal environment
Enriched Environment	Enriched environment improves motor function in intact and unilateral dopamine-depleted rats; Jadavji et al. (2006)	NSCs, Endogenous	Rats in an enriched environment demonstrated behavioral amelioration and had a significantly greater amount of surviving dopaminergic neurons	The enriched environment consisted of a larger cage that had toys, which were changed weekly. The rats also received different food types along with their typical chow
Enriched Environment	Enriched environment elevates expression of growth associated protein-43 in the substantia nigra of SAMP8 mice; Yuan et al. (2018)	NSCs, Endogenous	Mice placed in an enriched environment demonstrated improved learning and memory retention	The enriched environment consisted of a larger cage with running wheels, toys, nesting material, and several tunnels
Enriched Environment	An Enriched Environment Ameliorates Oxidative Stress and Olfactory Dysfunction in Parkinson’s Disease with α-Synucleinopathy, (Wi et al., 2018)	NSCs, Endogenous	The study demonstrated that enriched environments ameliorate olfactory dysfunction, oxidative stress, and decreased nitrated α-syn density in the olfactory bulb	The enriched environment consisted of a bigger cage that contained toys, shelters, running wheels, tunnels, and other mice for social interactions
Enriched Environment	Environmental Enrichment Prevents Transcriptional Disturbances Induced by Alpha-Synuclein Overexpression; Wassouf et al. (2018)	NSCs, Endogenous	In the wild type mice, this experiment demonstrated that enriched environments upregulate neurotrophic factors. In the transgenic mice that were experiencing overexpression of *SNCA*, enriched environments ameliorated transcriptional disturbances in glial cells	The enriched environment consisted of larger cages that housed eight female mice. In these cages, there was plentiful bedding and nesting material, and contained objects with varying shape, color, and texture. The cages also contained tunnels, climbing cubes, and running wheels, and all these objects were rearranged to maintain novelty
Enriched Environment	Alterations of Nigral Dopamine Levels in Parkinson’s Disease after Environmental Enrichment and PACAP Treatment in Aging Rats; Jungling et al. (2021)	NSCs, Endogenous	Rats in an enriched environment in tandem with pituitary adenylate cyclase-activating polypeptide demonstrated enhanced dopaminergic neuron preservation	The enriched environment consisted of a larger cage with toys, tunnels, various rotating rods, with half of the toys being changed daily. After 5 weeks of this environment the rats were placed in a normal environment
Enriched Environment	Enriched environment promotes similar neuronal and behavioral recovery in a young and aged mouse model of Parkinson’s disease; Goldberg et al. (2011)	NSCs, Endogenous	The study demonstrated a significant recovery of dopaminergic neurons in mice treated with 1-methyl-4-phenyl-1,2,3,6-tetrahydropyridine (MPTP)	Young adult and aged male mice were placed in an enriched environment which consisted of a large cage with toys that were being cycled every 3 days. Each cage housed 8–10 mice
Exercise and Stem Cell Transplantation	Exercise Promotes Neurite Extensions from Grafted Dopaminergic Neurons in the Direction of the Dorsolateral Striatum in Parkinson’s Disease Model Rats; Torikoshi et al. (2020)	NSCs, Endogenous and	Exercise after stem cell transplantation significantly enhanced the survival of dopaminergic neurons	Utilized rat model in this experiment. Transplantation alone demonstrated that four out of six grafts were able to survive, while the addition of exercise increased that number to five out of six
Exercise and Stem Cell Transplantation	Physical exercise and human adipose-derived mesenchymal stem cells ameliorate motor disturbances in a male rat model of Parkinson’s disease; Cucarián et al. (2019)	MSCs, Cerebral Injection (Striatum)	The study demonstrated in 6-OHDA lesioned rats that exercise resulted in enhanced behavioral amelioration. The synergistic effect of exercise and the stem cell implant group was negligible for motor function	The MSCs were derived from abdominal adipose tissue. The exercise consisted of progressive aerobic treadmill training
Exercise and Enriched Environment	The use of commercially available games for a combined physical and cognitive challenge during exercise for individuals with Parkinson’s disease—a case series report; Pradhan, (2019)	N/A (Study investigated exercise and behavioral outcome)	This study demonstrated that active video games can be a form of both enriched environment and exercise, and contribute to amelioration of typical motor deficiencies for patients with PD. Two participants experienced improved functional reach scores, while the third participant had a clinically important improvement on their 6MWT.	This study noted the importance of finding a suitable game for the target motor deficiency, evidenced by the lack of improvement in gait speed and single leg stance, which the participants did not have a game for
Exercise and Enriched Environment	Physical activity and environmental enrichment regulate the generation of neural precursors in the adult mouse substantia nigra in a dopamine-dependent manner; Klaissle et al. (2012)	NSCs, endogenous	Exercise and EE saw increased growth of adult neurons in substantia nigra	The experiment had a control with two mice in a standard cage, one group with an exercise wheel and two mice in a cage, and a third group with at least 5 mice and access to toys, food, and places to hide. Experiment did not examine simultaneous intervention

### 3.3 Enriched Environment/Exercise and Huntington’s Disease

The benefits of EE conditions for HD were first discovered in transgenic HD mice models during a landmark study in 2000. Van Dellen et al. found that HD mice exposed to stimulating environments demonstrate delayed cerebral atrophy and motor coordination compared to non-stimulated HD controls. Mice in the non-stimulated group also developed seizures, whereas the enriched group did not (van Dellen et al., 2000). Cognitive deficits in learning and memory, which typically precede motor manifestations in HD, were improved in the environmentally enriched group, specifically for task-specific flexibility and long-term spatial memory (Nithianantharajah et al., 2008). Furthermore, it is known that HD vastly affects intracellular signaling, transcriptional regulation, and protein expression, including downregulated BDNF expression (Cha, 2000; Spires, et al., 2004; Ferrer, et al., 2000). Environmental enrichment ameliorates the motor manifestations in HD and increases BDNF levels in the striatum and hippocampus, posing positive effects on neurogenesis and cell survival in this neurodegenerative disease (Spires et al., 2004; Lazic, et al., 2006). Like EE, PE demonstrates cognitive and motor benefits in rodent HD models (Pang et al., 2006). When spatial memory was assessed in HD mice by observing running alternations in a T maze, 45% of HD mice housed with running wheels alternated during the task, compared to only 29% of HD mice in standard housing. There was no significant difference in alternation rates between WT and standardized housing HD rodents, supporting that physical exercise can rescue cognitive function in HD. Decreased mRNA BDNF levels were also noted in the striatum, hippocampus, and anterior cortex of HD rodents, supporting dysregulated transcription. However, PE did not affect BDNF protein expression in either WT or HD groups. Running did increase striatal mRNA in this study, which the authors attribute to increased astrocytes secreting BDNF (Pang et al., 2006). Another study tested the long-term effects of PE on improving cognitive reserve in mice models, which interestingly revealed reduced escape latencies and longer survival in mice trained with cognitive stimulation compared with the only motor-trained group (Wood et al., 2011). Taken together, both environmental enrichment and physical exercise prove beneficial for ameliorating cognitive function, protein expression, and motor performance in HD (See Table 5).

**TABLE 5:** Experimental studies for Huntington's disease with stem cell transplantation, an enriched environment, exercise, and a combination of enriched environment or exercise with stem cells.

Huntington’s disease				
Type of intervention	Title, Author, Year	Stem cell	Significant findings	Other notes
Environmental Enrichment	Delaying the onset of Huntington’s in mice; van Dellen et al. (2000)	N/A (behavioral study)	Transgenic R6/1 HD mice exposed to stimulating environments (containing cardboard, paper, plastic objects) demonstrated delayed cerebral atrophy and motor coordination compared to non-HD controls	Exposure to enriched environments did not significantly affect spontaneous motor activity or body mass in both groups. Additionally, seizures were observed in the control group, but none in transgenic HD mice
Environmental Enrichment	Gene-environment interactions modulating cognitive function and molecular correlates of synaptic plasticity in Huntington’s disease transgenic mice; Nithianantharajah et al. (2008)	N/A (behavioral study and investigated post-synaptic markers)	R6/1 HD mice demonstrated impaired short and long-term spatial learning and memory. Environmental enrichment enhanced spatial learning in HD mice which employed improved spatial search strategies compared to the non-EE HD group	Significantly increased levels of hippocampal postsynaptic density protein 95 (PSD-95), which modulates post-synaptic signalling in excitatory neurons, were increased in EE-exposed HD mice compared to the non-EE group
Environmental Enrichment	Environmental Enrichment Rescues Protein Deficits in a Mouse Model of Huntington’s Disease, Indicating a Possible Disease Mechanism; Spires et al. (2004)	N/A (investigated striatal, cortical, and hippocampal tissue)	BDNF levels are reduced in the striatum of non-enriched mice. Conversely, this is rescued by environmental enrichment	There is unchanged BDNF expression in the anteromedial cortex of HD mice, suggesting that HD alters BDNF transport from the cortex to the striatum, rather than BDNF expression. Compared to non-enriched mice, this effect was rescued in the enriched group
Environmental Enrichment	Neurogenesis in the R6/1 transgenic mouse model of Huntington’s disease: effects of environmental enrichment; Lazic et al. (2006)	Neural progenitor cells in the hippocampal dentate gyrus	Investigators used BrdU (indicator of neural proliferation) and DCX (microtubule associated protein and indicator of neurogenesis) to mark NPCs in the hippocampal dentate gyrus. Older mice housed with EE conditions for 21 weeks had increased BrdU + cells compared to the non-EE group. Enrichment also ameliorated structural deficits in older HD mice, resulting in increased neuronal migration and longer neurites	Environmental enrichment had no significant effect on younger HD mice housed for 6 weeks, compared to older HD mice housed for 21 weeks
Exercise	Differential effects of voluntary physical exercise on behavioral and brain-derived neurotrophic factor expression deficits in Huntington’s disease transgenic mice; Pang et al. (2006)	N/A (behavioral study and quantified BDNF expression)	Voluntary physical exercise, specifically running, rescues cognitive deficits in HD mice which develop by 14 weeks before motor impairment	Running increased BDNF mRNA levels in the striatum, but not in the anterior cortex or hippocampus
Exercise and environmental enrichment	“Brain training” improves cognitive performance and survival in a transgenic mouse model of Huntington’s disease; Wood et al. (2011)	N/A (behavioral study)	Transgenic R6/2 mice were exposed to three stimulating environments combining different modes of stimulation and exercise: physical exercise (via the Rotarod), cognitive stimulation (OX maze), and mixed social stimulation and exercise (playground environment). Male HD mice trained with cognitive stimulation (OX maze) had reduced escape latencies compared with the other training groups. Only female Rotarod stimulated HD mice performed better on motor tasks, but this did not significantly affect cognitive performance. Playground-stimulated mice improved motor performance in both sexes, but had no significant impact on cognitive performance	Exposure to OX maze training improved both cognitive performance and survival in male mice, which was not apparent in the two other modes of stimulation
Exercise and striatal grafts	Associative plasticity in striatal transplants; Brasted et al. (1999)	Graft tissue from the whole ganglionic eminence of embryonic day 15 (E15) rat embryos	Rats with striatal lesions that received striatal grafts showed improved performance, with similar results as non-HD controls, during a lateralized discrimination task	Motor training ipsilateral to the lesion did not confer additional benefits, whereas training the contralateral side resulted in marked recovery. Note ipsilateral lesions affect the contralateral side, thus targeted training is vital for functional recovery
Exercise, environmental enrichment, and striatal grafts	The effects of lateralized training on spontaneous forelimb preference, lesion deficits, and graft-mediated functional recovery after unilateral striatal lesions in rats (Döbrössy and Dunnett, 2006b)	Graft tissue from the whole ganglionic eminence of E15 rat embryos	HD rats with striatal lesions trained to perform a food retrieval task with the ipsilateral paw performed similarly as non-HD controls. However, motor deficits in rats trained on the paw contralateral to the lesion and graft were rescued by graft transplantation	This study supports the role of targeted training, and use-dependent recovery
Exercise, environmental enrichment, and striatal grafts	Morphological and cellular changes within embryonic striatal grafts associated with enriched environment and involuntary exercise (Döbrössy and Dunnett, 2006a)	Graft tissue from the whole ganglionic eminence of E15 rat embryos	HD rats exposed to EE had increased striatal BDNF levels, increased graft spinal densities, and larger cell volumes compared to the exercise group	Suggests that surrounding environmental factors influence neural plasticity post-transplantation

### 3.4 Conclusion

EE and exercise show distinct regenerative effects that may work synergistically with stem cell-derived neural progenitor therapy if used in combination and following certain guidelines with timing, dosage, and intensity. Altogether, the use of these rehabilitation strategies may produce the most impact by potentially training the immature transplanted cells to form functional circuitry with the host’s neural networks leading to improved neuroanatomical graft-host integration and behavioral outcomes.

## 4 The Role of Enriched Environment and Exercise in Enhancing Neural Circuitry Repair With Stem Cells

**FIGURE 3:**
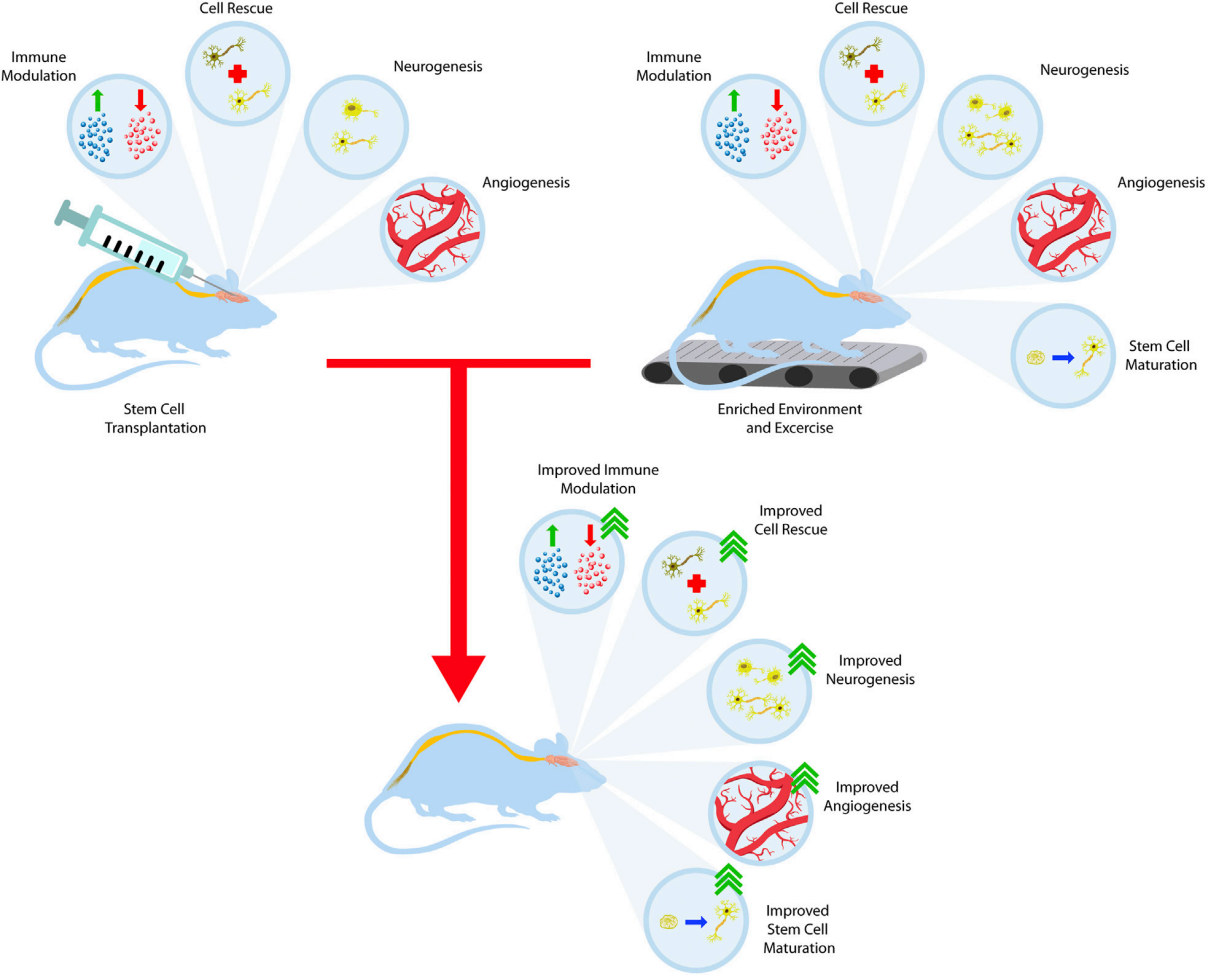
Training the stem cell graft to integrate with the host brain. Stem cell transplantation offers many therapeutic benefits such as immune system modulation, dying cell rescue, neurogenesis, and angiogenesis. When combined with the benefits of EE and exercise, these outcomes synergize and lead to better outcomes.

Effective stem cell therapy not only hinges on cell replacement but necessitates appropriate functional integration, connectivity, and differentiation into the host environment. Utilizing environmental enrichment and exercise to facilitate this process is an active field of research of great therapeutic value for stroke, PD, and HD. This concept centers around stimulating the graft site through EEs to enhance neuroplasticity and recruit endogenous repair mechanisms that favor survival, growth, and functional integration for transplanted cells (Döbrössy and Dunnett, 2005; Dunnett, 2013; Clinch et al., 2017). However, in the case of PD, restoring dopamine levels alone is able to ameliorate parkinsonian symptoms. The positive benefits from both EE and stem cell transplantation can further be discussed through combination studies rather than the individual applications of both stem cells and EE (Figure 3).

### 4.1 The Role of Enriched Environment and Exercise in Enhancing Neural Circuitry Repair With Stem Cells in Stroke

Studies using a preclinical stroke model with a combination of stem cells and rehabilitation have consistently revealed the importance of using a combination therapy when assessing outcomes. BDNF is a neurotrophic factor correlated with neural plasticity that has been well studied in the context of motor rehabilitation and stem cell transplantation. It has been proposed that rehabilitation strategies that enhance BDNF also enhance motor recovery after ischemia (Mang et al., 2013). Because stem cells and rehabilitation have been shown to increase this neurotrophic factor, it can be theorized that the combination of both therapies will have an additive effect on functional outcome (Ploughman et al., 2009; Mang et al., 2013). In section 2, we assessed the current theories of stem cell mechanisms for brain repair. Indeed, stem cells lead to enhanced recovery through many means, but the biobridge paves a new avenue for understanding the amelioration of deficits after stroke. The increases in endogenous NPCs must be met with increased training to lead to functional integration of the grafts with the host tissue. Rehabilitation offers the means by which grafts mature and integrate effectively. However, the type of rehabilitation method is an important factor. Aerobic exercise has been shown to increase BDNF and endogenous neurogenesis (Mang et al., 2013). Forced low-intensity exercise increases NPC maturation and facilitates a more robust motor recovery (Morishita et al., 2020). Most importantly, both EE and stem cell transplantation increase adult neurogenesis which has been implicated as the main driving force behind recovery (Zhan et al., 2020; Garthe et al., 2016; Tajiri et al., 2103; Singhal et al., 2014; van Praag et al., 1999) The stem cells induce neuroprotection early and influence proliferation when the rodents are still in recovery. Once the rodents begin to display functional recovery, EE should be implemented quickly to increase neurogenesis and neuronal integration into the host tissue. The timing and implementation of exercise and EE remains questionable. Exercise, if implemented too early, results in increased thalamic atrophy and worse functional motor outcomes and should be further evaluated in combination studies (Kozlowski et al., 1996; Risedal et al., 1999). EE, byways of housing enrichment, is safe and effective when implemented early or late in the disease course and may not pose the same problems (Tang et al., 2019; Zhan et al., 2020).

The three-phase paradigm might combat this careful balancing effect between exploiting the positive results and mitigating adverse effects based on timing and the intervention needed (Zhan et al., 2020). Treadmill exercise enhances the therapeutic potency of MSCs by increasing the survival of existing neurons and transplanted neurons (Zhang et al., 2015). In addition, combined therapy led to reduced infarct volume, increased synaptogenesis, and overall improved behavioral outcomes compared to lesioned rats treated with either stem cells or rehabilitation (Akhoundzadeh et al., 2017). The timing for implementation between the stem cells and the rehabilitation strategy needs more data to find a balance accurately, as many studies show differences in timing and results. There is believed to be a defined plastic window in which neural plasticity can be exploited for better outcomes after a pathologic event or process (Hara, 2015; Zhan et al., 2020).

### 4.2 The Role of Enriched Environment and Exercise in Enhancing Neural Circuitry Repair With Stem Cells in Parkinson’s Disease

**FIGURE 4:**
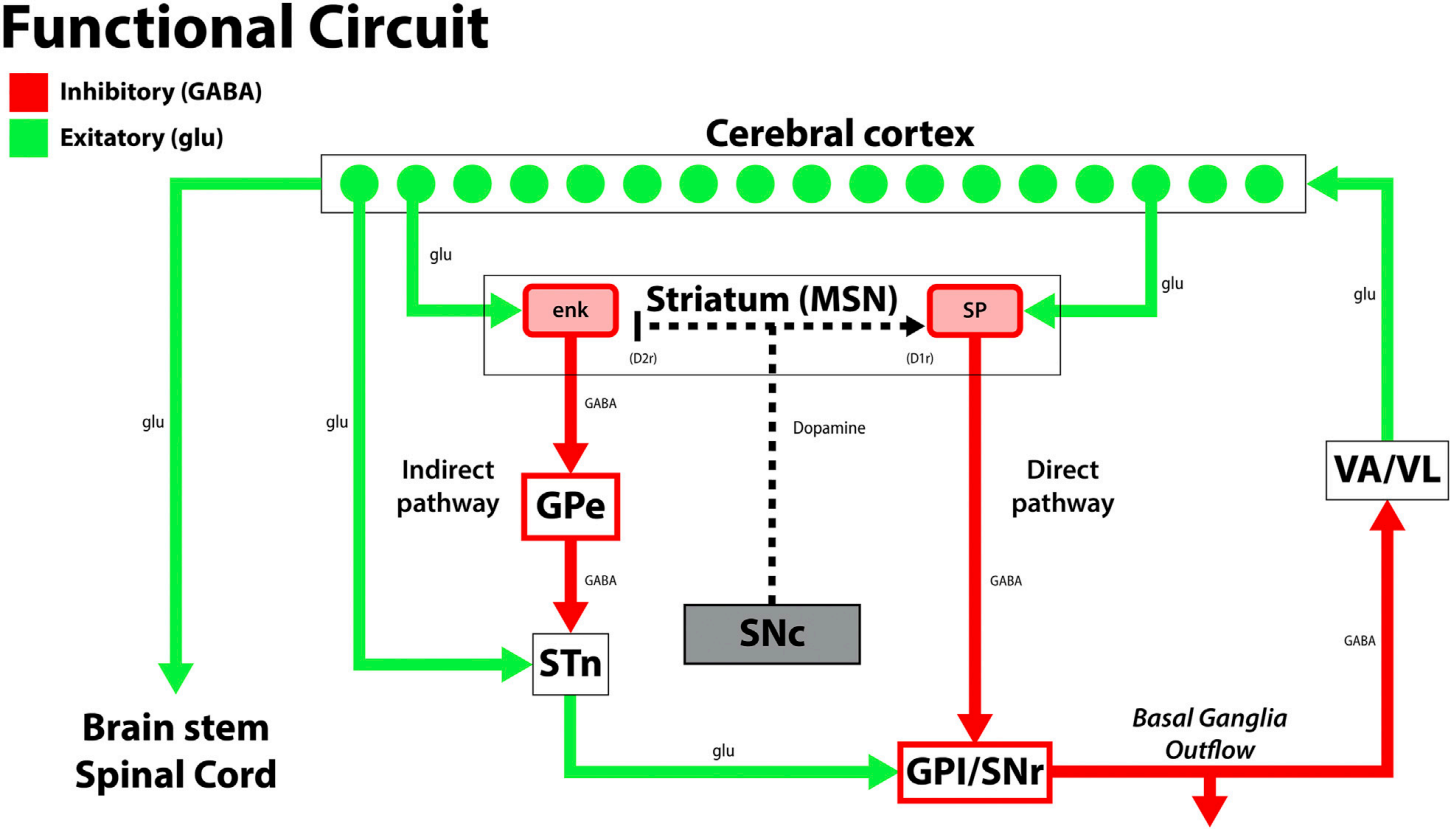
A diagram of the classical and simplified functional circuit of the basal ganglia. Dopaminergic neurons from the substantia nigra pars compacta (SNc) project to the medium spiny neurons (MSNs) of the striatum and modulate the direct and indirect pathway activity to modulate inhibition by the thalamus (VA/VL). Green arrows signify glutamatergic excitatory neurons and red arrows signify GABA expressing inhibitory neurons. enk, enkephalin; SP, Substance P; GPe, globus pallidus, external segment; GPi, globus pallidus, internal segment; SNc, substantia nigra pars compacta; SNr, substantia nigra pars reticulata; STN, subthalamic nucleus; VA/VL, ventral anterior/ventral lateral nucleus of the thalamus; D1r, D1 dopaminergic Gs coupled receptor; D2r dopaminergic Gi coupled receptor. Adapted from (McGregor and Nelson, 2019).

Exercise induces morphological changes in toxin-induced PD rat models. When dopaminergic grafts from the rat fetal ventral mesencephalon were transplanted into the striatum of 6-OHDA lesioned rats, rodents exposed to exercise had enhanced dopaminergic graft survival, maturation, and neurite extension into the dorsolateral striatum. These neurite extensions specifically grew in the same pathway taken by A9 dopaminergic neurons, which project into the striatum and are crucial for improved behavior (Figure 4). This is an important finding as A9 dopaminergic neurons preferentially degenerate in PD (Björklund and Dunnett, 2007; Torikoshi et al., 2020). Additionally, hemispheric dominance plays an influential role in dopaminergic neuroplasticity post-transplantation. Rats unilaterally lesioned with 6-OHDA were observed in skilled forelimb experiments pre- and post-DA graft placement. Rats that did not demonstrate a paw preference during the staircase test improved the most post-transplantation. Subjects with skilled movements contralateral to the lesion recovered moderately whereas those with ipsilateral lateralization recovered the least (Nikkah et al., 2001). Clinically, exercise-induced neuroplasticity plays a profound impact on long-term rehabilitation in PD patients. 8 weeks of balance training improved functional mobility and reduced falls in PD patients for up to 12 months following treatment, and Tai Chi training for 12–24 weeks reduced falls for up to 6 months post-training (Li et al., 2012; Gao et al., 2014; Shen and Mak, 2015; Wong-Yu and Mak, 2015; Mak et al., 2017). Furthermore, patients with early PD who practiced intensive treadmill exercise had improved postural control and increased striatal dopaminergic graft binding to the dopaminergic receptor, D2R, as confirmed by PET imaging. These changes were not observed in the non-exercise PD group (Fisher et al., 2013).

### 4.3 The Role of Enriched Environment and Exercise in Enhancing Neural Circuitry Repair With Stem Cells in Huntington’s Disease

A seminal study by Brasted and colleagues revealed the importance of targeted training to recover lost motor function in HD rat models. These animals were specifically trained to perform a lateralized choice reaction task before receiving quinolinic acid injections into the neostriatum and retrained for 30 days to perform the same task with or without a striatal graft. After retraining post-recovery, only rats that received striatal grafts showed marked performance and eventually achieved similar results as non-HD controls. Additionally, motor training ipsilateral to the lesion did not confer additional benefits, whereas training the contralateral side resulted in marked recovery. Since ipsilateral lesions affect the contralateral side, this supports that rehabilitative training specific and targeted to the lesion is vital for functional recovery in HD (Brasted, et al., 1999). Further investigation into post-graft recovery with lateralized training showed that HD rats with striatal lesions trained to perform a food retrieval task with the ipsilateral paw performed similarly as non-HD controls, whereas those trained on the paw contralateral to the lesion showed motor deficits that recovered with graft transplantation (Döbrössy and Dunnett, 2006b). Döbrössy and Dunnett also investigated changes in BDNF levels, dendritic spinal densities, and cell volume in rodent HD models subjected to EE and exercise, post-striatal graft transplantation (Döbrössy and Dunnett, 2006a). The HD group exposed to EEs in the form of several cardboard tunnels, ladders and platforms revealed increased BDNF levels, spinal densities, and cell volume. In contrast, decreased spinal densities and BDNF were found in the exercise group, suggesting that surrounding environmental factors influence neural connectivity and plasticity post-transplantation (Döbrössy and Dunnett, 2006a).

### 4.4 Conclusion

Stem cell therapy not only relies on cell replacement, but also necessitates successful graft-host functional integration. Enhancing graft integration through rehabilitative strategies advances current treatment options for stroke and HD. This concept may not be as important for PD as replacement of dopaminergic neurons and the presence of dopamine ameliorates the symptoms. EE and exercise facilitate stem cell graft-host reconstruction of neural circuitry that may involve at least a two-pronged mechanism that creates a conducive microenvironment into the host brain, allowing the newly transplanted cells to survive, proliferate, and differentiate into neural cells. Both may additionally train the transplanted immature cells to learn the neurochemical, physiological, and anatomical signals in the brain towards better functional graft-host connectivity.

## 5 Summary and Future Directions

Limited therapeutic options exist for stroke, PD, and HD, prompting further research into their underlying pathology to improve functional recovery and slow disease progression. Regenerative medicine, specifically stem cell therapy, is a promising avenue that consistently improves outcomes in preclinical studies. This is an active field of research with underlying mechanisms that include cell replacement, endogenous stem cell recruitment, neurotrophic effects, and induction of anti-inflammatory properties. While replacing damaged neurons is the first step to functional recovery, grafts must integrate, differentiate, and enhance synaptic plasticity to maintain and promote healing. EE and exercise combined with stem cell therapy achieve this via enhanced synaptogenesis, neuronal survival, axonal regrowth, dendrite extension, and enhanced receptor binding. There is a continued need for clinical trials that demonstrate the rehabilitative effects of combined EE, exercise, and stem cell therapy. Further research is also required to elucidate appropriate timing between stem cell transplantation and rehabilitation. Ultimately, understanding how these rehabilitative strategies facilitate graft integration and neural repair encourages a team-based and multi-faceted approach to treating these diseases and is core to developing robust therapeutic options for patients across the globe.
